# Impact of Nicotine-Free Electronic Cigarettes on Cardiovascular Health: A Systematic Review

**DOI:** 10.3390/jcm14248717

**Published:** 2025-12-09

**Authors:** Ivana Jukic, Tina Becic, Ivona Matulic, Petra Simac, Jonatan Vukovic

**Affiliations:** 1 Department of Internal Medicine, Division of Gastroenterology, University Hospital of Split, 21000 Split, Croatia; jonatan.vukovic@mefst.hr; 2 Faculty of Health Sciences, University of Split, Ulica Rudjera Boskovica 35, 21000 Split, Croatia; 3 Cardiovascular Disease Department, University Hospital of Split, 21000 Split, Croatia; 4 Private Clinic Matulic, Osjecka Ulica 24a, 21000 Split, Croatia; ivonamatulic@yahoo.com; 5 Department of Internal Medicine, Division of Rheumatology, Allergology and Clinical Immunology, University Hospital of Split, 21000 Split, Croatia; petra_simac@hotmail.com; 6 Internal Medicine Department, School of Medicine, University of Split, Soltanska 2, 21000 Split, Croatia

**Keywords:** nicotine-free e-cigarettes, cardiovascular risk, oxidative stress, endothelial dysfunction, systematic review, vascular health

## Abstract

**Background**: The cardiovascular effects of nicotine-containing e-cigarettes are well-established; however, far less is known about nicotine-free formulations, which are often perceived as safer alternatives. Yet, these products generate complex aerosols that may still pose toxicological risks. **Methods**: Following the PRISMA 2020 guidelines, we systematically searched PubMed, Scopus, Web of Science, and the Cochrane Library up to 27 August 2025. Eligible studies included human and animal research on nicotine-free e-cigarettes evaluating vascular, hemodynamic, arrhythmic, oxidative, or inflammatory outcomes. Owing to the heterogeneity of the studies, the findings were synthesized qualitatively. **Results**: Nine studies met the inclusion criteria. Human trials consistently demonstrated acute vascular impairments, including endothelial dysfunction, oxidative stress, increased arterial stiffness, and transient blood pressure elevations. Experimental models corroborated these findings and further revealed systemic inflammation, mitochondrial injury, and developmental cardiotoxicity. **Conclusions**: Nicotine-free e-cigarettes produce reproducible acute cardiovascular effects. Although the long-term outcomes remain uncertain, preclinical evidence highlights biologically plausible mechanisms, including mitochondrial dysfunction and proarrhythmogenic potential. Until large-scale longitudinal studies have been conducted to clarify their risk profile, nicotine-free products should not be regarded as risk-free.

## 1. Introduction

In recent years, the use of electronic cigarettes (e-cigarettes, EC) has grown steadily, reflecting changes in nicotine consumption across the population. Although originally promoted as an alternative to conventional tobacco products for smokers, ECs are now commonly used by adolescents, young adults, and both current and former smokers [1,2]. Among adolescents and young adults, ECs are often perceived as less harmful than conventional tobacco products and, in some cases, as a safer or even ‘harmless’ alternative. Such perceptions have been associated with an increased likelihood of experimentation and continued use in this age group [3,4,5]. Extensive research has examined the cardiovascular and systemic effects of nicotine-containing e-cigarettes (NCECs), demonstrating increased sympathetic activity, increased heart rate, oxidative stress, and evidence of vascular dysfunction, whereas the health impact of nicotine-free (NF) variants remains largely unexplored [6,7,8]. The use of ECs is concerning because they expose users to complex aerosolized mixtures. Along with the base solvents—propylene glycol (PG) and vegetable glycerin (VG)—EC liquids typically include a wide range of synthetic flavoring agents, many of which have not been adequately tested for inhalation safety and can also introduce particulate matter such as metals and silicates [9,10,11]. Recent MRI-based studies in healthy volunteers have also shown transient vascular dysfunction following nicotine-free e-cigarette (NFEC) use [12].

Emerging evidence indicates that nicotine-free e-cigarette aerosols are not biologically inert. During heating, PG and VG can undergo thermal degradation, generating highly reactive carbonyl compounds such as formaldehyde, acetaldehyde, and acrolein. These aldehydes have been implicated in vascular toxicity through mechanisms involving oxidative stress, endothelial injury, protein adduct formation, and pro-inflammatory signaling cascades [13,14,15]. These compounds also activate redox-sensitive transcription factors such as NF-κB, leading to the downstream production of cytokines and adhesion molecules that contribute to vascular inflammation.

Flavoring chemicals such as diacetyl, cinnamaldehyde, and vanillin have been detected in a wide range of EC products and are of particular concern due to their toxicity. Experimental studies demonstrate that these compounds impair mitochondrial function, reduce ATP production, increase mitochondrial ROS (mtROS) formation, and induce cytotoxicity in vascular endothelial cells—even in the absence of nicotine [16,17]. Disruption of mitochondrial homeostasis may lead to opening of the mitochondrial permeability transition pore (mPTP), which further amplifies oxidative stress and promotes apoptotic signaling. Similarly, developmental cardiotoxicity has been observed in zebrafish embryos exposed to NFEC e-liquids, suggesting a potential impact on early cardiovascular development [18].

Evidence from human studies, although limited in scope, supports these concerns. Acute exposure to NFEC aerosols has been associated with transient increases in arterial stiffness, impaired flow-mediated dilation, and markers of systemic oxidative stress in healthy volunteers [19,20,21].

Animal studies further support the plausibility of cardiovascular toxicity, reporting endothelial dysfunction, lipid peroxidation, and inflammatory infiltration following prolonged exposure to flavored NFEC vapors [22,23]. Notably, the absence of nicotine does not eliminate the pro-oxidant and pro-inflammatory potential of EC aerosols. Clinical magnetic resonance imaging (MRI) data further indicate that acute exposure to NF aerosols can transiently increase the aortic stiffness in healthy adults [24].

In murine models, exposure to NFEC aerosols triggers mitochondrial dysfunction, excessive ROS production, and endothelial barrier disruption, through mechanisms involving glycocalyx shedding—an early event in vascular injury associated with impaired mechanotransduction and enhanced vascular permeability [25,26].

A growing body of evidence also highlights potential electrophysiological disturbances. Aldehydes such as acrolein can impair myocardial conduction, disrupt calcium handling, and alter ion-channel function, thereby creating a substrate for arrhythmogenesis even in nicotine-free environments. Experimental models demonstrate prolonged ventricular repolarization, increased susceptibility to triggered activity, and impaired synchronization of myocardial contraction—findings that underscore the potential for the proarrhythmogenic effects of NFEC aerosols through oxidative and inflammatory pathways.

Although evidence of harm from non-nicotine constituents of EC liquids continues to grow, regulatory oversight remains limited. Existing frameworks—such as the European Union’s Tobacco Products Directive (TPD) and the U.S. Food and Drug Administration’s (FDA) regulations on electronic nicotine delivery systems (ENDSs)—are largely centered on nicotine content and product labeling, with far less emphasis on solvents, flavorings, and other non-nicotine ingredients. Consequently, products marketed as ‘nicotine-free’ may create a misleading perception of safety among consumers [27,28,29].

Combustible cigarettes remain orders of magnitude more harmful than e-cigarettes across cardiopulmonary outcomes, and several population-based and biomarker studies report lower inflammation, oxidative stress, and cardiovascular disease incidence among exclusive e-cigarette users compared with smokers [30]. A prospective analysis of Population Assessment of Tobacco and Health (PATH) data showed that exclusive e-cigarette users had a significantly lower incidence/onset of cardiovascular disease and myocardial infarction than people who smoked cigarettes; exclusive e-cigarette users were no different from non-users [31]. A systematic review of 63 studies showed that switching from cigarettes to e-cigarettes resulted in significant reductions in blood flow and blood pressure; moreover, vapers who never smoked cigarettes did not differ from non-users [32].

The objective of this systematic review is to isolate the cardiovascular effects attributable specifically to nicotine-free e-cigarette aerosols (propylene glycol/vegetable glycerin, with or without flavorings). This risk-focused lens does not contradict harm-reduction principles; rather, it clarifies which hazards may persist in the absence of nicotine and, therefore, merit regulation, labeling, and targeted research. Given the increasing prevalence of NFEC use and the lack of comprehensive evidence-based assessments of their cardiovascular safety, there is a clear need for a systematic synthesis of the available literature. Previous reviews have either focused exclusively on nicotine-containing devices or included NFEC products only tangentially, without isolating their specific effects.

This systematic review aims to critically evaluate the current human and animal evidence on the cardiovascular impact of NFECs, with a focus on the endothelial function, arterial stiffness, blood pressure, heart rate, arrhythmias, oxidative stress, and inflammatory responses Throughout this review, findings from nicotine-free aerosols are considered separately from those involving nicotine-containing products in order to avoid conflating distinct exposure profiles.

## 2. Materials and Methods

### 2.1. Literature Search Strategy

A systematic literature search was conducted to identify studies evaluating the cardiovascular effects of NFEC. Four electronic databases were searched from their inception to 27 August 2025: PubMed/MEDLINE, Scopus, Web of Science Core Collection, and the Cochrane Library (CENTRAL). This systematic review was conducted in accordance with the Cochrane Handbook and adhered to the Preferred Reporting Items for Systematic Review and Meta-Analyses (PRISMA) guidelines to ensure the appropriate quality of the assessment (Supplementary Table S1). The search strategy combined controlled vocabulary and free-text terms, as follows: PubMed: (“electronic cigarette*” OR “e-cigarette*” OR vaping) AND (“nicotine-free” OR “non nicotine” OR “without nicotine”) AND (“cardiovascular” OR “vascular” OR “blood pressure” OR “endothelium” OR “oxidative stress” OR “inflammation”); Scopus/Web of Science/Cochrane Library: same search terms adapted to database-specific syntax and field tags. Filters were applied to limit the results to English-language peer-reviewed original studies involving human or animal models. We used Boolean operators and database-specific syntax to refine the search strategy, aiming to balance the sensitivity and specificity across the databases.

Only studies published in English were considered. Filters were applied to limit the results to original articles involving human or animal models. Editorials, commentaries, reviews, and in vitro-only studies were excluded. There were no restrictions on the publication date. Additional studies were identified by manually screening the reference lists of the included articles and searching preprint repositories and clinical trial registries. We defined nicotine-free e-cigarettes (NFEC) as PG/VG-based aerosols labeled 0 mg/mL nicotine. Studies using THC/cannabinoids, caffeine, vitamin additives (e.g., vitamin E acetate), or other psychoactives were excluded.

**Figure 1:**
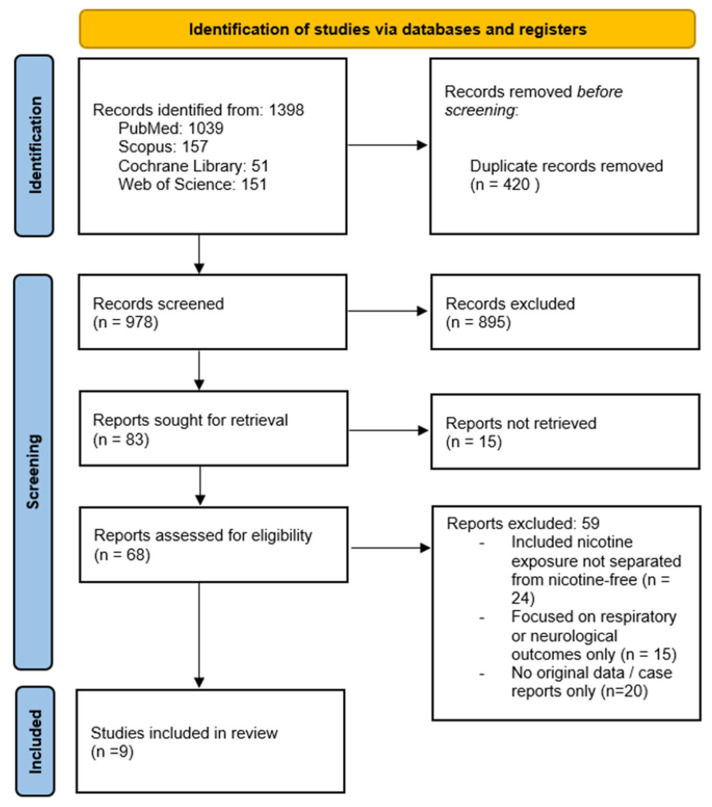
PRISMA 2020 flow diagram of study selection. From 1398 records, 978 were screened, and 68 full texts assessed. Fifty-nine reports were excluded for not meeting the criteria, leaving nine studies in the qualitative synthesis.

All records were imported into Zotero (Zotero [Computer software]; Version 6.0; Corporation for Digital Scholarship: Fairfax, VA, USA, 2024. Available at https://www.zotero.org/) for deduplication and screening. Two reviewers (T.B. and I.M.) independently screened the titles and abstracts, followed by a full-text assessment of potentially eligible studies. Discrepancies were resolved through discussion. The overall study selection process is illustrated in the PRISMA 2020 flow diagram (Figure 1). The review was written in accordance with the PRISMA guidelines and registered on the Open Science Framework (OFS) https://doi.org/10.17605/OSF.IO/ZFJ3V). The protocol outlines the objectives, search strategy, and inclusion criteria for evaluating the effects of nicotine-free e-cigarettes on the cardiovascular system.

### 2.2. Risk of Bias Assessment

Two reviewers (T.B. and I.M.) independently evaluated the risk of bias in all the included studies. Randomized controlled trials were assessed using the Cochrane RoB2 tool, non-randomized clinical studies using ROBINS-I, and animal or embryonic models using the SYRCLE tool adapted for experimental research [33]. Any disagreements were resolved by consensus. A summary of the overall risk of bias judgments is presented in Table 1.

### 2.3. Data Synthesis

Given the heterogeneity of the included studies in terms of design (human trials, animal models, zebrafish embryos), populations (healthy volunteers vs. experimental models), and outcomes (MRI-based vascular stiffness, flow-mediated dilation, oxidative stress biomarkers, developmental toxicity), a quantitative meta-analysis was not feasible. Instead, we conducted a structured qualitative synthesis. The findings are presented narratively and grouped by study design (human, animal, experimental) and type of cardiovascular outcome assessed. The included studies are discussed in the text and are presented in Table 2.

## 3. Results

### 3.1. Qualitative Synthesis

The search identified 1398 records across four databases (PubMed = 1039, Scopus = 157, Web of Science = 151, Cochrane Library = 51). After removing 420 duplicates, 978 titles and abstracts were screened. Of these, 895 were excluded, leaving 83 for full-text retrieval. A total of 15 studies could not be obtained, and 68 were assessed in detail. Fifty-nine were excluded for reasons outlined in the PRISMA flow diagram (Figure 1), resulting in nine eligible studies. Of these, six were conducted in humans and three in animal or embryonic models.

### 3.2. Animal and Embryonic Models

**Figure 2:**
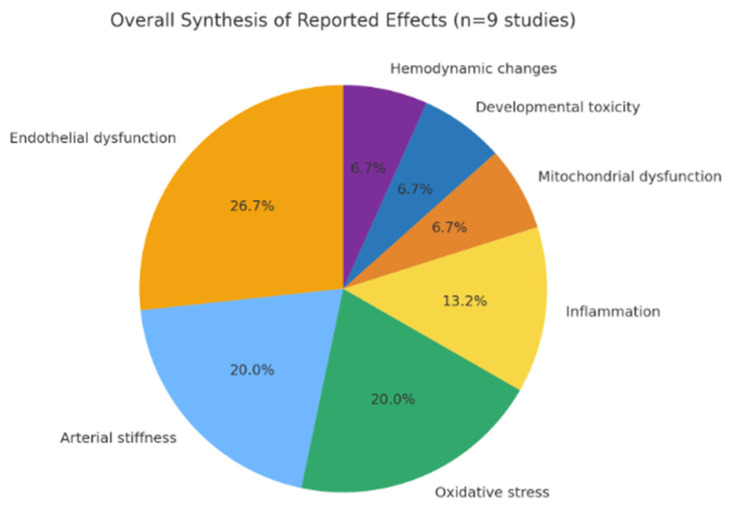
Overall synthesis of reported effects, showing that NFECs most often caused endothelial dysfunction, arterial stiffness, and oxidative stress, with less frequent reports of inflammation, mitochondrial dysfunction, developmental toxicity, and hemodynamic changes.

The findings from animal and embryonic models were largely consistent with human evidence, while also providing mechanistic insights. Zebrafish embryos exposed to high-dose NFEC aerosols exhibited developmental cardiotoxicity [18]. In murine models, chronic NFEC exposure promoted systemic inflammation and vascular oxidative stress as well as mitochondrial dysfunction and disruption of the endothelial barrier [34,35]. Collectively, these studies suggest that oxidative stress, inflammation, and mitochondrial injury represent central biological pathways underlying the cardiovascular toxicity of NFEC aerosols. Table 2 summarizes the study designs, exposures, main outcomes, and limitations across the human and experimental models [18,26,28,29,30,34,35,36]. Figure 2 provides an overview of the main cardiovascular outcomes reported across the studies, illustrating the frequency and direction of the observed effects (e.g., transient increases in arterial stiffness, oxidative stress markers, or endothelial dysfunction). In contrast, Table 2 presents detailed study-level information, including the design, exposure duration, aerosol composition, and key methodological limitations. Together, these visual summaries allow for comparison of acute versus repeated exposure findings while maintaining transparency in study heterogeneity.

### 3.3. Human Studies

Across the six available human studies, a consistent pattern indicated that NFEC aerosols acutely impair vascular function. The reported effects included transient oxidative stress and endothelial dysfunction, reduced flow-mediated dilation, and increased arterial stiffness accompanied by systemic inflammation in randomized trials [12,21,22,23,24,25,26,31]. It is important to note that several studies, including those by Chaumont et al. and Franzen et al., reported minimal or non-significant vascular effects following exposure to nicotine-free aerosols, highlighting inconsistency across the evidence base [19,20].

Advanced MRI studies have further demonstrated that exposure to NFEC aerosols can acutely increase the aortic stiffness [12,34]. More recently, a crossover trial found rises in the central blood pressure and augmentation index, although these effects were smaller and shorter in duration than those observed in NCEC products [34,35]. Collectively, the evidence suggests that even without nicotine, EC aerosols produce consistent adverse effects on vascular and hemodynamic function. It is important to note, however, that these investigations were small, of short duration, and primarily conducted in young healthy adults.

### 3.4. Risk of Bias

Overall, the nine included studies demonstrated a moderate risk of bias (Table 1). The randomized trial conducted by Chaumont et al. in 2018 was of generally good methodological quality but limited by incomplete blinding [19]. Non-randomized clinical studies were more prone to moderate performance and detection bias, mainly due to small sample sizes, single-center settings, and potential confounding. In contrast, animal and embryonic studies often lacked sufficient reporting of randomization and blinding, resulting in unclear to moderate risk. The risk of bias assessment (Table 1) identified predominantly unclear or high risk in several key domains, particularly regarding the exposure quantification, blinding, and randomization. These methodological weaknesses reduce confidence in the reported short-term physiological effects and limit the strength of causal inferences. Minor labeling inconsistencies identified in the initial version of the table have been corrected in the revision.

## 4. Discussion

The emerging evidence from both clinical and experimental studies indicates that exposure to NFECs is not biologically inert. Given the small number of available studies and their considerable methodological heterogeneity, the findings presented here should be interpreted as preliminary signals rather than definitive evidence of cardiovascular harm. The evidence indicates that oxidative stress represents a central pathogenic mechanism through which nicotine-free aerosols impair cardiovascular function. Elevated levels of reactive oxygen species (ROS), lipid peroxidation products, and reductions in nitric oxide (NO) bioavailability have been consistently demonstrated in both human and experimental models, suggesting early redox imbalances independent of nicotine exposure. In healthy volunteers, acute inhalation of NFEC aerosols was shown to impair vascular function, as measured by reductions in flow-mediated dilation and tissue oxygen saturation [12]. While some human studies observed transient vascular changes after NFEC exposure, others detected no significant effects, underscoring the variability and uncertainty in the current findings.

In vitro work further demonstrated that e-liquids, particularly those containing certain flavoring agents, can promote oxidative stress, trigger endothelial dysfunction, and polarize macrophages toward a pro-inflammatory phenotype even in the absence of nicotine [35]. To ensure conceptual clarity, the mechanistic and clinical findings involving nicotine-containing formulations were not interpreted as direct evidence for nicotine-free aerosols unless explicitly demonstrated. Several mechanistic studies not included in the primary synthesis are referenced to provide biological context; these should be regarded as supporting evidence rather than direct findings from the nine included studies. The thermal degradation of PG/VG generates aldehydes such as acrolein and formaldehyde, which directly disrupt endothelial homeostasis by forming protein adducts, reducing endothelial nitric oxide synthase (eNOS) activity, and activating redox-sensitive transcription factors, including NF-κB. These processes promote endothelial dysfunction and initiate inflammatory signaling cascades relevant to early atherogenesis. Recent mechanistic work also highlights degradation of the endothelial glycocalyx as an early event, following exposure to nicotine-free aerosols. Shedding of glycocalyx components disrupts the vascular barrier integrity, impairs mechanotransduction, and increases the vulnerability to oxidative and inflammatory injury, providing a biologically plausible link to the transient vascular dysfunction observed in human studies. NFEC aerosols have also been shown to activate multiple inflammatory pathways, including upregulation of IL-6, TNF-α, ICAM-1, and VCAM-1, promoting leukocyte adhesion and vascular inflammation. These immunological responses align with observed increases in systemic oxidative stress and endothelial impairment, suggesting a coordinated inflammatory phenotype driven by non-nicotine constituents. Additional studies and systematic reviews suggest that NF formulations may disrupt mitochondrial homeostasis, leading to membrane depolarization, excess reactive oxygen species generation, and energy depletion [26,36]. These mechanistic observations originate from external preclinical sources and are presented solely to contextualize potential pathways relevant to NFEC exposure. Experimental studies further support mitochondrial dysfunction as a key mediator of NFEC-induced cardiovascular toxicity. Aerosol constituents have been shown to impair mitochondrial respiration, increase mitochondrial ROS production, and promote opening of the mitochondrial permeability transition pore (mPTP), thereby triggering apoptotic pathways and compromising cellular energy metabolism. Developmental toxicity has also been reported, with prenatal exposure to NFEC aerosols in mice resulting in reduced birth weight and craniofacial malformations [28]. While some investigations have failed to observe significant adverse effects in the absence of nicotine, the overall body of evidence supports a more cautious interpretation of NFECs than is commonly assumed.

### 4.1. Comparison with the Existing Literature

The evidence from human studies offers valuable, although sometimes inconsistent, insights into the acute vascular impact of EC aerosols. Carnevale et al. (2016) reported that brief exposure to EC aerosols, both with and without nicotine, increased oxidative stress and impaired endothelial function in healthy individuals [21]. In a randomized crossover trial, Chaumont et al. (2018) found that EC aerosols containing nicotine impaired vascular function and elevated oxidative stress, whereas the NF vehicle produced no significant changes in endothelial or arterial measures [19]. Using advanced imaging techniques, Caporale et al. (2019) observed acute reductions in flow-mediated dilation and modest increases in arterial stiffness after inhalation of NFEC aerosols [12]. Similarly, Papaioannou et al. (2019) and Meng et al. (2023) identified transient alterations in vascular parameters following NFEC use, although the clinical relevance of these findings remains uncertain [24,31].

The vascular effects of NFEC aerosols are biologically plausible because heating e-liquid constituents such as PG and glycerin produces reactive aldehydes (formaldehyde, acrolein) and other volatile organic compounds that can impair the redox balance and endothelial integrity [13,31]. Experimental work also indicates that certain flavoring agents may amplify these effects [14,16]. Practices that increase the coil temperature beyond optimal ranges, such as dripping liquid directly onto heated coils, have been linked to disproportionately high aldehyde yields [37,38].

Animal and embryonic models add important mechanistic depth. Piechowski et al. (2021) demonstrated developmental cardiovascular toxicity in zebrafish embryos exposed to cinnamon-flavored EC aerosols, underscoring the risks during early development [18]. Espinoza-Derout and colleagues reported systemic inflammation, vascular oxidative stress, and atherosclerotic changes in murine models following e-cigarette exposure [23]. Dai et al. (2025) combined in vivo and in vitro experiments to show that NFEC aerosols impair mitochondrial function, increase reactive oxygen species, and disrupt endothelial barrier integrity [25]. In rats, chronic exposure did not significantly change the blood pressure or heart rate overall, but aged animals exhibited evidence of diastolic dysfunction [26,39]. Narrative reviews further link EC use to pro-atherogenic pathways—including endothelial dysfunction, oxidative stress, and inflammation—supporting biological plausibility for atherosclerosis and thrombosis [26,39,40,41,42]. Flavorant-driven mitochondrial injury has also been shown in lung epithelial cells, reinforcing the potential systemic relevance of these mechanisms [26].

Taken together, these findings suggest that NFEC aerosols can activate some of the same oxidative and inflammatory stress pathways identified in studies on NCEC use [6,7,8]. Importantly, adverse effects have also been observed outside the cardiovascular system, such as compromised gastrointestinal epithelial barrier integrity and heightened inflammation [41].

### 4.2. Arrhythmias and Blood Pressure Effects

#### Narrative Synthesis

Most human NFEC studies have concentrated on vascular function and arterial stiffness; however, there is growing evidence that they may also influence cardiac rhythm and hemodynamics.

Preclinical studies provide the clearest evidence for the arrhythmogenic potential of NFEC aerosols. In murine models, the inhalation of propylene glycol and vegetable glycerin alone has been shown to trigger ventricular premature beats, slow cardiac conduction, and prolong ventricular repolarization. Although data on atrial arrhythmias are more limited, chronic exposure experiments indicate an increased vulnerability to both atrial and ventricular arrhythmias when the heart is stressed [42]. In addition, thermal degradation products such as acrolein have been found to disrupt myocardial synchrony and impair contractile performance in mice, supporting a biologically plausible proarrhythmogenic pathway even without nicotine [42,43].

In clinical studies, direct reports of arrhythmias following acute NFEC exposure are lacking. Instead, most trials have focused on vascular and hemodynamic responses. Caporale et al. (2019) and Papaioannou et al. (2019) both observed impaired endothelial responses and transient increases in arterial stiffness, pointing to early vascular vulnerability [12,24]. Blood pressure findings have been mixed: Franzen et al. (2018) reported no increase in systolic pressure after NFEC exposure, whereas NCEC devices produced sustained rises [20]. In contrast, Papaioannou et al. (2019) and Meng et al. (2023) found transient increases in both systolic and diastolic pressure that resolved within two hours [24,31]. Other work has shown that acute EC inhalation can elevate oxidative stress biomarkers and alter the autonomic balance [44]. Recent meta-analyses confirm these inconsistencies, noting that NCEC products consistently raise the systolic pressure, while the effects of NFECs are weaker, less predictable, and typically short-lived [45,46]. A comparative summary of the cardiovascular findings in both human and animal studies is listed in Table 3.

Overall, the evidence from both human and animal studies indicates that NFECs are not physiologically inert. Clinical data consistently demonstrate acute vascular dysfunction, oxidative stress, and variable hemodynamic responses, while animal models point to deeper mechanisms involving oxidative stress, mitochondrial injury, and—based on the findings reported by Carll et al. (2022), Qiu et al. (2022), and Thompson et al. (2017, 2019)—disturbances in electrophysiological stability [36,40,42,47]. A broad review of the literature further confirms the cardiovascular risks across populations and device types [44]. Taken together, these findings strengthen the biological plausibility of cardiovascular harm and highlight the need for larger, longer-term clinical studies.

### 4.3. Interpretation

Interpreting the results, particular attention was paid to the methodological quality of the studies included. By integrating the risk of bias assessment with the outcome synthesis, we observed that the lower-bias human studies consistently demonstrated endothelial dysfunction and transient arterial stiffness following NFEC exposure, whereas the higher-bias experimental studies primarily contributed mechanistic insight. This integrated interpretation underscores that while the current evidence supports the biological plausibility of cardiovascular effects, the overall certainty remains moderate due to study-level methodological constraints.

The available evidence makes it increasingly clear that NFECs cannot be considered biologically inert. Human studies consistently demonstrate acute disturbances in vascular and hemodynamic function, while animal and cellular models reveal complementary mechanisms involving oxidative stress, inflammation, mitochondrial injury, and—in supporting evidence—a potential for arrhythmia. Oxidative stress appears to be a particularly central pathway, linking aerosol exposure with vascular dysfunction and inflammatory activation [31,44]. More recently, mechanistic studies have shown that NFEC aerosols not only impair mitochondrial function and generate ROS but also compromise endothelial barrier integrity [26,38]. Persistent mitochondrial dysfunction from vaping has been implicated in chronic inflammation across systems [48,49,50,51]. Authoritative reviews further reinforce these mechanistic insights, underscoring that oxidative stress, vascular dysfunction, and autonomic imbalance are convergent pathways through which ECs can promote cardiovascular harm [52].

The absence of documented arrhythmias in human trials likely reflects methodological limitations rather than genuine safety. Although human data remain limited, preclinical models indicate that oxidative and aldehyde-mediated injury may extend to electrophysiological disturbances. Altered calcium handling, impaired ion-channel function, prolonged repolarization, and increased susceptibility to triggered activity have been documented, supporting a potential proarrhythmogenic effect of nicotine-free aerosols that warrants systematic evaluation in future clinical studies. Taken together, these findings emphasize the need for larger, longer-term clinical investigations that go beyond vascular endpoints to include the systematic assessment of rhythm disturbances and underlying molecular pathways, in order to better define the cardiovascular risks of NFEC use. Recent systematic reviews reinforce these concerns, highlighting reproducible associations between e-cigarette use and adverse vascular outcomes, although the specific contribution of NFECs remains less clearly defined [44,45,46,50,51].

### 4.4. Strengths and Limitations

A key limitation of this systematic review is the heterogeneity of the included studies. The evidence base for this review remains limited, comprising only nine studies with considerable methodological heterogeneity in terms of the exposure duration, study design, and outcome measures. As such, these findings should be interpreted as exploratory and hypothesis-generating rather than confirmatory. Most available studies assess acute physiological responses that may not directly reflect long-term cardiovascular risk. Transient changes in arterial stiffness, oxidative stress, or endothelial function can occur after various stimuli such as caffeine intake or physical exertion; therefore, their clinical relevance remains uncertain. These acute effects, however, provide mechanistic insights and should be viewed as early biological signals warranting further longitudinal investigation. Interpretation of the current evidence must also consider the generally high or unclear risk of bias across the included studies. Incomplete blinding and inconsistent exposure characterization may have introduced systematic errors or overestimated the magnitude of acute responses. These limitations emphasize the need for standardized experimental protocols and objective exposure verification in future work. Some clinical trials in humans evaluated the vascular function and arterial stiffness using standardized protocols, whereas others relied on animal or embryonic models that investigated different endpoints such as developmental toxicity or mitochondrial dysfunction. This variability prevented us from pooling the results into a formal meta-analysis. While the absence of quantitative synthesis limited the ability to provide precise effect estimates, the qualitative approach allowed us to capture the breadth of available evidence and to identify consistent patterns of vascular dysfunction and oxidative stress across study designs. Findings from animal models should be interpreted with caution, as exposure conditions in experimental settings often exceed those encountered in typical human use. Such high-dose or prolonged exposures are valuable for elucidating the mechanisms of oxidative stress and inflammation but may not directly predict clinical outcomes in humans. The lack of observed long-term cardiovascular effects in current human studies could therefore reflect both methodological limitations and genuine interspecies differences in susceptibility or exposure dynamics.

Most of the human studies were also relatively small, short-term, and conducted at a single center, often enrolling young and otherwise healthy volunteers. These characteristics limit the generalizability of the findings to broader populations, particularly older adults or those with cardiovascular risk factors, who may be more susceptible to harm. In addition, rhythm outcomes were not systematically assessed, which likely accounts for the lack of documented arrhythmias in clinical trials despite supportive evidence from animal studies.

At the same time, the review has several notable strengths. We conducted a comprehensive search across multiple databases, applied independent risk of bias assessments using validated tools (RoB 2, ROBINS-I, and SYRCLE), and adhered to the PRISMA 2020 guidelines to ensure transparency and rigor. Together, these steps enhance the reliability of the review and provide a balanced summary of the current state of knowledge.

These limitations collectively restrict the generalizability of the findings and underscore the need for standardized exposure protocols and adequately powered longitudinal studies.

### 4.5. Clinical and Public Health Implications

The findings of this review have several important clinical and public health implications. From a regulatory perspective, NFECs are often subject to less stringent oversight than NC products; however, a growing body of evidence suggests that this regulatory gap may be difficult to justify. Marketing these devices as “safe” or “harmless” risks misleading consumers and could encourage uptake among adolescents and non-smokers [49]. For clinicians, it is important to emphasize that “nicotine-free” does not mean “risk-free.” Although switching from combustible tobacco to ECs may lead to short-term improvements in vascular function, the durability of these effects and the long-term cardiovascular consequences remain uncertain [52]. Recent population-based data further suggest a modestly elevated risk of cardiovascular disease associated with EC use [53].

Adolescent use is a particular concern. In the United States, national surveillance data showed a marked rise in EC use among youth between 2014 and 2018 [1]. Moreover, EC use during early adolescence has been linked to an increased likelihood of using combustible tobacco [53]. Similar trends have been documented internationally, with steady increases in prevalence among youth and young adults over the past decade [2]. From a research standpoint, there is an urgent need for longitudinal human studies that track chronic cardiovascular outcomes, including blood pressure regulation, arrhythmias, and structural vascular changes. Advanced imaging techniques and biomarker-based approaches may help connect acute physiological changes with the development of long-term cardiovascular disease. Evidence from acute exposure studies further suggests that NFEC aerosols can impair vascular function and oxygen delivery, raising concerns about potential systemic consequences over time [12,31].

Given the limited evidence, any regulatory or clinical implications should be considered tentative, and further research is needed before firm policy recommendations can be formulated. In summary, the current evidence indicates that NFECs can trigger acute cardiovascular effects and activate toxicological pathways similar to NCEC products. Their long-term impact, however, remains uncertain, highlighting the need for rigorous large-scale longitudinal studies to define their risk profile more clearly.

## 5. Conclusions

The current evidence base is limited and heterogeneous; therefore, the conclusions of this review should be interpreted with appropriate caution. This systematic review suggests that nicotine-free e-cigarettes may induce acute biologically plausible changes in vascular and oxidative parameters; however, the current evidence is insufficient to determine the long-term cardiovascular risk. These findings highlight the need for well-controlled longitudinal and mechanistic studies rather than supporting specific regulatory actions at this stage. Unlike prior reviews that combined nicotine-containing and nicotine-free exposures, our approach is novel in that it isolates the cardiovascular effects attributable specifically to nicotine-free aerosol constituents.

Human studies consistently show acute endothelial dysfunction, impaired vascular reactivity, increased arterial stiffness, and modest transient elevations in blood pressure, while animal and experimental models provide mechanistic support by revealing oxidative stress, mitochondrial injury, systemic inflammation, and developmental cardiotoxicity. Importantly, although arrhythmias have not yet been documented in clinical trials, the preclinical evidence clearly indicates proarrhythmogenic potential, conduction disturbances, and impaired repolarization, underscoring the need for the systematic evaluation of rhythm outcomes in future human research. While these effects are largely transient in the context of short-term human exposure, the absence of long-term data prevents any reliable assessment of whether repeated or chronic NFEC use may contribute to hypertension, arrhythmias, or structural cardiovascular disease. Given the increasing prevalence of NFEC use, particularly among adolescents and young adults, who are most susceptible to misleading perceptions of safety, stronger regulatory oversight is urgently warranted. Public health communication must explicitly emphasize that “nicotine-free” does not equate to “risk-free.” By isolating the effects of NFEC, this review provides an essential foundation for evidence-based risk communication, regulatory action, and future research priorities. Future research should prioritize large-scale longitudinal human studies that move beyond acute vascular endpoints to incorporate standardized measures of blood pressure regulation, arrhythmia surveillance, vascular remodeling, and advanced imaging modalities. Mechanistic investigations into oxidative stress, mitochondrial dysfunction, and inflammatory signaling pathways will also be critical to connect acute physiological disturbances with long-term cardiovascular outcomes. In conclusion, NFECs cannot be regarded as harmless alternatives to conventional tobacco or nicotine-containing e-cigarettes. Until robust longitudinal evidence clarifies their cardiovascular risk profile, regulatory authorities, clinicians, and public health practitioners should adopt a precautionary stance, ensuring that consumers are fully informed of their potential harm. These conclusions should be interpreted within the context of the current evidence limitations. While some studies have emphasized potential cardiovascular risks, others point to the substantially lower overall toxicity of e-cigarettes compared with combustible tobacco. Ultimately, this review does not seek to endorse or condemn any product, but to provide an objective synthesis that supports an evidence-based policy and informs public health decisions. Until more robust long-term human data become available, interpretations regarding public health or regulatory relevance should remain cautious and provisional.

## Figures and Tables

**Table 1:** Risk of bias assessment of the included studies.

Study	Model	Tool	Selection Bias	Performance Bias	Detection Bias	Reporting Bias	Overall Risk
Piechowski et al., 2021 [18]	Zebrafish (animal)	SYRCLE	Unclear	Moderate	Moderate	Low	Moderate
Espinoza-Derout et al., 2021 [23]	Mouse (animal)	SYRCLE	Unclear	Moderate	Moderate	Low	Moderate
Dai et al., 2025 [25]	Mouse + cells (animal)	SYRCLE	Unclear	Moderate	Moderate	Low	Moderate
Carnevale et al., 2016 [21]	Human (crossover)	ROBINS-I	Low	Moderate	Moderate	Low	Moderate
Franzen et al., 2018 [20]	Human (clinical)	ROBINS-I	Low	Moderate	Moderate	Low	Moderate
Chaumont et al., 2019 [27]	Human (RCT)	RoB2	Low	Low	Moderate	Low	Low–Moderate
Caporale et al., 2019 [12]	Human (MRI)	ROBINS-I	Low	Moderate	Moderate	Low	Moderate
Papaioannou, 2019 [24]	Human (MRI)	ROBINS-I	Low	Moderate	Moderate	Low	Moderate
Goebel et al., 2023 [28]	Human (crossover)	ROBINS-I	Low	Moderate	Moderate	Low	Moderate

Note. Risk of bias was assessed using the Cochrane RoB2 tool for randomized controlled trials, ROBINS-I for non-randomized human studies, and the SYRCLE tool for animal and experimental models. Most studies were judged to have a moderate overall risk of bias, mainly due to the small sample sizes, short-term interventions, and incomplete reporting of blinding and randomization.

**Table 2:** Summary of included studies, with study designs, exposures, main outcomes, and limitations across human and experimental models. Upward arrows (↑) in the table means indicates that elements increased, downward (↓) decreased.

Author	Year	Model	Exposure	Main Findings	Limitations
Piechowski et al. [18]	2021	Animal (zebrafish embryos)	High-dose NFEC aerosols infused into dechlorinated water	Decreased end systolic and diastolic volume, stroke volume, heart rate, cardiac output, and red blood cell density	Experimental, high dose only
Espinoza-Derout et al. [23]	2021	Animal (mouse)	Short-term exposure	Hyperlipidemia, sympathetic dominance, DNA damage, and macrophage activation	Short exposure duration
Dai et al. [25]	2025	Animal (mouse + endothelial cells)	NFEC aerosol exposure	Dose-dependent increases in mitochondrial ROS production, enhanced endothelial permeability, and glycocalyx degradation	Translational relevance uncertain
Goebel et al. [28]	2023	Human (crossover, n = 17)	0 mg/mL e-cigarette (DIPSE-eGo, tobacco flavor)	↑ Central BP, ↑ augmentation index, and ↑ small airway resistance in all groups, with more pronounced effects with nicotine	Small sample, single-center, acute effects only
Carnevale et al. [21]	2016	Human (crossover trial)	Acute exposure to traditional tobacco vs. NFEC exposure	NFECs and traditional tobacco show comparative decreases in vitamin E levels and flow-mediated dilation	Small sample, short-term
Franzen et al. [20]	2018	Human (clinical study)	Acute exposure to tobacco, NCEF, and NFECs in healthy adults	↓ FMD in all groups; NFEC did not significantly alter BP or heart rate	Single-center, short-term
Chaumont et al. [27]	2019	Human (RCTs)	Fourth-generation EC exposure with and without nicotine	↓ transcutaneous oxygen tension in all groups, ↓ arterial oxygen tension, and ↑ airway epithelial injury following EC aerosol at high wattage with and without nicotine	Small sample size
Caporale et al. [12]	2019	Human (MRI study)	Acute EC aerosol exposure	↑ resistivity index, luminal FMD blunted, and reduced peak velocity; aortic pulse wave marginally increased	Healthy volunteers only
Papaioannou [24]	2019	Human (MRI study)	NFEC exposure	↑ Aortic stiffness, as measured by aortic pulse wave velocity, and impaired endothelial function	Limited generalizability

**Table 3:** Comparative summary of cardiovascular findings in human and animal studies. Upward arrows (↑) in the table means indicates that elements increased, downward (↓) decreased.

Model/Study	Key Findings
Animal/embryonic studies	
Piechowski et al. (2021) [18]	Developmental cardiotoxicity in zebrafish embryos exposed to high-dose nicotine-free aerosols.
Espinoza-Derout et al. (2021) [23]	Systemic inflammation and vascular oxidative stress in mice after short-term exposure.
Dai et al. (2025) [25]	Mitochondrial dysfunction, ↑ ROS, and endothelial barrier disruption in murine and cell models.
Carnevale et al. (2016) [21]	↑ Oxidative stress and endothelial dysfunction after acute exposure to nicotine-free aerosols.
Supporting evidence (not included in primary synthesis)	
Carll et al. (2022) [47]	Nicotine-free aerosol constituents induced atrial/ventricular arrhythmias, slow conduction, and repolarization defects in animal models.
Human studies	
Franzen et al. (2018) [20]	↓ Flow-mediated dilation following nicotine-free vaping.
Chaumont et al. (2019) [27]	↑ Arterial stiffness and systemic inflammation in RCT of nicotine-free exposure.
Caporale et al. (2019) [12]	MRI evidence of ↑ aortic stiffness after acute nicotine-free aerosol inhalation.
Papaioannou (2019) [24]	Transient ↑ aortic stiffness in healthy volunteers.
Goebel et al. (2023) [28]	↑ Central BP, ↑ augmentation index, and ↑ small airway resistance, though less pronounced than with nicotine products.

Note. This table summarizes the main cardiovascular effects of nicotine-free e-cigarette exposure across the nine studies included in the systematic review (six human and three animal/embryonic). Carll et al. (2022) [47] is presented as supporting evidence only and was not part of the primary synthesis.

## Supplementary Materials

The following supporting information can be downloaded at https://www.mdpi.com/article/10.3390/jcm14248717/s1, Table S1: PRISMA 2020 checklist [54].

## Abbreviations

The following abbreviations are used in this manuscript:

BP	Blood pressure
ENDS	Electronic nicotine delivery system
FDA	Food and Drug Administration
FMD	Flow-mediated dilation
MRI	Magnetic resonance imaging
NC	Nicotine containing
NF	Nicotine free
NFEC	Nicotine-free e-cigarettes
PG	Propylene glycol
RCT	Randomized controlled trials
ROS	Reactive oxygen species
TPD	Tobacco Products Directive
VG	Vegetable glycerin
